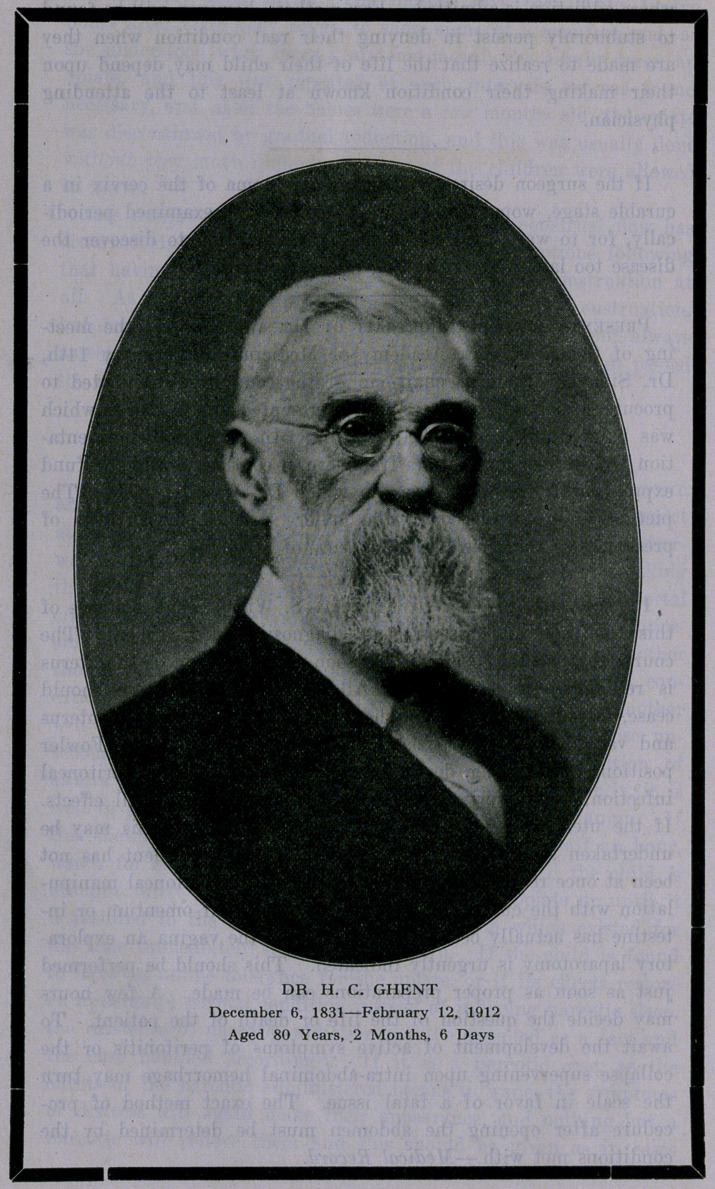# Dr. H. C. Ghent

**Published:** 1912-03

**Authors:** 


### EDITORIAL DEPARTMENT

**DR. F. E. DANIEL, Editor.** **DR. R. H. L. BIBB, Associate Editor.**

### DR. H. C. GHENT.

Another of the Texas Fathers in Medicine has fallen; another of the old regime,—of the aristocracy of medicine long dominant in the South,—has passed away and gone to his reward. He is now with the shades of our beloved Swearingen, Cupples, Ashbel Smith, Becton, Starley, Wallace—in the Elysian fields.

We shall miss him. At our annual meetings we shall miss his cordial handshake, his manly, earnest face, his genial smile and his wise counsel. Peace to his ashes!

Dr. Ghent was no ordinary man. He was a strong man, earnest in the cause of his beloved profession, to which he had given fifty-eight years of his life, a strenuous one,—a life of strong will and earnest endeavor; yet he died—a poor man! He was a humanitarian, an altruist. Like all strong men who have their convictions, and the courage to defend them, he had many enemies; but it must be a poor soul indeed that can go through life battling for right who does not make enemies. But, where he had one enemy, he had scores of warm, attached and admiring friends. They knew him, understood him, appreciated him. His death is a loss not only to the community where he lived and labored so long and earnestly, and efficiently, but to the medical profession and to the people of the State. The poor of Bell county have lost a friend in need, a friend in deed.

Dr. Ghent was born in Laurens District, South Carolina, December 6, 1831. Married October 6, 1864, to Miss Sarah Jane Pearce, of Talladega county, Alabama. He came to Texas, January 6, 1866, locating at Port Sullivan, in Milam county, and engaged in the practice of medicine, and removed to Belton in October, 1873. In 1872, while the State was still laboring under the blighting and withering influence of radical rascality and misrule, he was sent to the Legislature by the people of Milam county as their Representative, and rendered efficient service in the redemption of the State. Dr. Ghent was a fluent writer and an earnest and eloquent speaker, and took an active interest in public affairs, being a strong and loyal Democrat.

The following is an epitome of his record:

M. D., Jefferson Medical College, class of 1856; honorary degree, University of Louisville; pupil of Gross, Miller, Rogers, Flint, and Stephen Smith, 1854-'6; delegate from Texas to International Medical Congress, 1885; member American Medical Association, and its Vice-President in 1885; President Bell County Medical Society, 1888; President Texas State Medical Association, 1884; President Central Texas District Association, 1887-'9, and 1890.

Dr. Ghent is survived by his wife and several daughters, the eldest being the wife of Professor Marvin L. Graves of the Texas State Medical College at Galveston.

## Figures and Tables

**Figure f1:**